# Indirubin improves antioxidant and anti-inflammatory functions in lipopolysaccharide-challenged mice

**DOI:** 10.18632/oncotarget.17560

**Published:** 2017-05-02

**Authors:** Tianjie Qi, Haitao Li, Shuai Li

**Affiliations:** ^1^ Department of Respiratory Medicine, The Second Hospital of Hebei Medical University, Shijiazhuang, Hebei 050000, P.R. China

**Keywords:** indirubin, acute lung injury, oxidative stress, inflammation, NF-κB

## Abstract

Indirubin, a traditional Chinese medicine formulation from the Muricidae family, has been reported to exhibit abroad anti-cancer and anti-inflammation activities and mediate nuclear factor-κB (NF-κB) signal. Thus, this study aimed to investigate the protective effects of indirubin on LPS-induced acute lung injury and the potential mechanism in mice. The results showed that LPS treatment caused oxidative stress and inflammation in mice. Indirubin alleviated LPS-caused oxidative stress and inflammation via reducing MDA abundance and IL-1β and TNF-α expressions in mice. Meanwhile, indirubin improved lung NO production and inhibited NF-κB activation caused by LPS exposure. In conclusion, indirubin alleviated LPS-induced acute lung injury via improving antioxidant and anti-inflammatory functions, which might be associated with the NO and NF-κB signals.

## INTRODUCTION

Indirubin, a traditional Chinese medicine formulation from the Muricidae family, has been demonstrated to affect physiological and pathophysiological processes, such as cell proliferation and death [1, 2]. Currently, indirubin has been considered to be a strong promise for clinical anticancer activity and also be useful in other diseases, such as Alzheimer's disease and diabetes [3]. Anti-inflammatory function and immune mediation of indirubin have been identified in various models. For example, indirubin ameliorates dextran sulfate sodium-induced ulcerative colitis in mice through the inhibition of inflammatory response and the induction of regulatory T cells [4]. In lipopolysaccharide (LPS)-induced mastitis mouse model, indirubin improves inflammation via inhibiting the production of interleukin (IL)-1β, IL-6, and tumor necrosis factor-α (TNF-α) [5].

Nuclear factor-κB (NF-κB), a transcription factor of inflammatory cytokines, has been widely demonstrated to involve in cellular responses to various stress, such as oxidative stress and infection [6, 7]. While various reports suggest that indirubin also can influence NF-κB signal [5, 8], which further mediates inflammatory response. Thus, indirubin can be served as a potential anti-inflammatory agent to treat inflammation-associated diseases. Acute lung injury is a major causes of acute respiratory failure characterized by oxidative stress, inflammatory response, and immune suppression [9, 10]. While the mechanism of acute respiratory failure and protection strategies are not full investigated. Thus, in this study, we used LPS-induced acute lung injury to investigate the protective role of indirubin in lung inflammation and the potential mechanism.

## RESULTS

### Effects of indirubin on LPS-induced lung wet/dry weight ratio in mice

As shown at Table 1, LPS treatment markedly increased lung wet/dry weight ratio (*p* < 0.05). Indirubin tended to reduce lung wet/dry weight ratio, but the difference was insignificant (*p* > 0.05).

**Table 1:** Effects of indirubin on LPS-induced lung wet/dry weight ratio in mice

Item	Control	LPS	LPS+ indirubin
Wet/dry weight ratio	6.21 ± 0.53^b^	9.93 ± 1.04^a^	8.36 ± 0.58^ab^

Data are expressed as the mean ± standard error of the mean. Values in the same row with different superscripts are significant (*P* < 0.05).

### Antioxidant function

In LPS-induced acute lung injury, glutathione peroxidase (GSH-Px) activity was markedly inhibited and malondialdehyde (MDA) abundance was significantly higher compared with the control group (Table 2), suggesting that LPS treatment caused lung oxidative stress (*p* < 0.05). Although indirubin failed to alleviate LPS-inhibited GSH-Px activity, indirubin markedly reduced MDA generation compared with the LPS group (*p* < 0.05).

**Table 2:** Indirubin alleviated oxidative stress in LPS-induced acute lung injury

Item	Control	LPS	LPS+ indirubin
GSH-Px (U/mL)	142.13 + 14.14^a^	101.23 ± 7.42^b^	126.32 ± 11.84^ab^
SOD (U/mL)	64.35 ± 6.23	54.04 ± 4.04	50.53 ± 6.24
CAT (U/mL)	6.57 ± 1.00	5.90 ± 1.02	5.86 ± 0.65
MDA (nmol/mL)	0.35 ± 0.05^b^	0.54 ± 0.11^a^	0.47 ± 0.07^b^

Data are expressed as the mean ± standard error of the mean. Values in the same row with different superscripts are significant (*P* < 0.05).

### Immunoglobulins (Igs)

Lung Igs (IgA, IgG, and IgM) were determined in this study and the results showed that LPS increased IgM and IgG levels (*p* < 0.05), while indirubin injection markedly reduced IgM abundance in the lung (*p* < 0.05) (Table 3).

**Table 3:** Effect of indirubin on Immunoglobulins (Igs) in LPS-induced acute lung injury (U/mL)

Item	Control	LPS	LPS+ indirubin
IgA	0.15 ± 0.02	0.12 ± 0.01	0.13 ± 0.02
IgM	0.57 ± 0.16^a^	0.76 ± 0.08^b^	0.69 ± 0.05^a^
IgG	0.99 ± 0.13^a^	1.31 ± 0.12^b^	1.06 ± 0.13^b^

Data are expressed as the mean ± standard error of the mean. Values in the same row with different superscripts are significant (*P* < 0.05).

### Nitric oxide synthase (NOS) activity

NOS activity was inhibited after exposure to LPS in mice (*p* < 0.05) (Table 4). Similarly, LPS also reduced nitric oxide (NO) generation and indirubin treatment improved NO generation (*p* < 0.05), suggesting that NOS/NO involved in the protective mechanism of LPS-induced acute lung injury.

**Table 4:** Effect of indirubin on NOS activity and NO in LPS-induced acute lung injury

Item	Control	LPS	LPS+ indirubin
NOS (U/ml)	10.15 ± 0.72^a^	8.12 ± 0.51^b^	9.13 ± 0.52^ab^
NO (nmol/ml)	0.57 ± 0.16^a^	0.36 ± 0.08^b^	0.49 ± 0.05^a^

Data are expressed as the mean ± standard error of the mean. Values in the same row with different superscripts are significant (*P* < 0.05).

### Inflammatory response

In this study, LPS treatment markedly induced inflammatory response in the lung evidenced by the upregulation of IL-1β, IL-6, and TNF-α (*p* < 0.05) (Table 5). Indirubin injection alleviated LPS-induced inflammation via decreasing IL-1β and TNF-α mRNA abundances in the lung (*p* < 0.05).

**Table 5:** Effect of indirubin on response in the lung in LPS-induced acute lung injury

Genes	Control	LPS	LPS+ indirubin
IL-1β	1.00 ± 0.09^b^	1.71 ± 0.27^a^	1.32 ± 0.16^b^
IL-6	1.00 ± 0.12^b^	1.27 ± 0.12^a^	1.36 ± 0.09^a^
IL-10	1.00 ± 0.19	0.97 ± 0.19	1.23 ± 0.18
TNF-α	1.00± 0.17^b^	1.50 ± 0.27^a^	1.17 ± 0.15^b^

Data are expressed as the mean ± standard error of the mean. Values in the same row with different superscripts are significant (*P* < 0.05).

### NF-κB

NF-κB widely involves in oxidative stress and inflammation. In this study, we found that NF-κB was markedly activated after LPS exposure in the lung (*p* < 0.05) (Table 6). As the upstream signal of NF-κB [11, 12], TLR4 and Myd88 were also determined and the results showed that LPS upregulated expression of TLR4 and Myd88 in the lung (*p* < 0.05). Meanwhile, indirubin markedly inhibited TLR4 and NF-κB signals, which might further mediate the oxidative stress and inflammation in the LPS-induced acute lung injury.

**Table 6:** Effects of matrine on expression of NF-κB

Item	Control	LPS	LPS+ indirubin
TLR4	1.00 ± 0.11^b^	1.55 ± 0.17^a^	1.14 ± 0.17^b^
Myd88	1.00 ± 0.14^b^	1.47 ± 0.13^a^	1.29 ± 0.15^ab^
NF-κB	1.00 ± 0.17^b^	1.51 ± 0.09^a^	1.20 ± 0.13^b^

Data are expressed as the mean ± standard error of the mean. Values in the same row with different superscripts are significant (*P* < 0.05).

## DISCUSSION

The goal of this study was to determine the protective effects of indirubin on LPS-induced acute lung injury and the potential mechanism in mice. The clinical index showed that LPS caused pulmonary edema evidenced by the increased lung wet/dry weight ratio. Indirubin tended to alleviate LPS-induced pulmonary edema in mice. Meanwhile, indirubin alleviated LPS-induced acute lung injury via improving antioxidant and anti-inflammatory functions.

Excess generation of free redical species and oxidative stress have been suggested to involve in the development of acute respiratory failure [13, 14]. To maintain cellular oxidative balance, antioxidant enzymes (i.e. GSH-Px, SOD, and CAT) are produced to reduce free redical species [15]. In this study, we found that indirubin failed to enhance the antioxidant function but significantly alleviated LPS-induced MDA production, a major lipid oxidative maker. These results indicated that indirubin alleviated lung oxidative stress in LPS-induced acute lung injury in mice. The antioxidant function of indirubin might contribute to the beneficial mechanism in the LPS-induced acute lung injury, as several reports have shown that oxidative injury occurs in LPS-induced acute lung injury and antioxidant therapy plays a beneficial role in LPS-induced acute lung injury [16, 17].

Dysfunction of NOS/NO exists in various pathological conditions, including inflammation and oxidative stress [18, 19]. In LPS-induced acute lung injury, NOS expression and NO production have altered in response to inflammatory response [20, 21]. In this study, indirubin improved lung NO production. NO is released into the blood circulation during sepsis, stimulating inflammatory cell recruitment and activation [22]. Meanwhile, NOS/NO also can involve in the activation of NF-κB siganl [23], which further mediates inflammation in LPS-induced acute lung injury.

Inflammatory response and immune suppression have been confirmed to involve in the progression and development of acute lung injury [17]. The present results exhibited that LPS induced inflammation and immune suppression in the lung via influencing IgM, IgG, IL-1β, IL-6, and TNF-α, while indirubin markedly reduced IgM abundances and IL-1β and TNF-α mRNA abundances in LPS-induced acute lung injury. Similarly, Kim et al. also found that indirubin alleviated serum Igs (IgE) production in 1-chloro-2,4-dinitrobenzene-induced skin inflammation [24]. Meanwhile, the anti-inflammatory function of indirubin has been widely identified in various models. In LPS-induced inflammatory response, indirubin inhibited inflammatory cytokines production via mediating NF-kB signaling pathway [5]. In addition, indirubin analogue (indirubin-3-monoxime) also exhibited anti-inflammatory effect via inhibiting the release of pro-inflammatory cytokines (IL-1β and IL-6) induced by LPS in RAW264.7 cells [25].

NF-κB mediates cytokines expression and involves in various inflammatory diseses, including LPS-induced acute lung injury [26, 27]. In this study, LPS upregulated expression of NF-κB and its upstream proteins (TLR4 and Myd88), while indirubin markedly alleviated NF-κB activation, which might serve as the protective mechanism in LPS-induced acute lung injury.

## MATERIALS AND METHODS

### Animal model and groups

Kunming mice (36 females) were purchased at 6–8 weeks of age and randomly assigned into 3 groups (*n* = 10): a control group, a LPS-challenged group, and a group in which mice given both indirubin and LPS. LPS (Sigma, St. Louis, MO, USA) was used to induce acute lung injury by i.p. injection of 15 mg/kg LPS according to previous report. Indirubin (Shanghai Yuan Ye Biological Technology Co., Ltd, Shanghai, China), dissolved in PBS (10:1), was was given by i.p. injection of at dose levels of 0.2 mL/20 g 1 hour before LPS treatment. All mice were sacrificed after 24 h and lung samples were harvested. This study was approved by the animal welfare committee of the Second Hospital of Hebei Medical University.

### Wet-to-dry lung weight ratio (W/D ratio)

The right lungs were obtained immediately weighed to get the wet weight. Then the lungs were placed at 80°C for 48 h to obtain the dry weight. The ratio of wet lung to dry lung was calculated to assess tissue edema.

### Oxidative stress

Lung samples were weighed and then homogenized in phosphate buffer (w/v: 1/9) on crushed ice using a tissue grinder. After centrifugation at 3500 g for 10 min at 4°C, the supernatant was collected for future use. GSH-Px, SOD, and CAT activity and MDA level in the lung homogenate were measured using spectrophotometric kits (Nanjing Jiangcheng Biotechnology Institute, China).

### NOS activity and NO determination

Lung NOS activity was detected using an ELISA kit according to the manufacturer's instructions (Shanghai Meilian Bio. Tech., China). Nitric oxide NO concentration were measured as released NO metabolites (nitrates and nitrites) using assay kits in accordance with the manufacturer's instructions (BiovisionInc.,USA).

### Immunoglobulins (Igs)

Lung Igs (IgA, IgG, and IgM) were determined by spectrophotometric kits (Nanjing Jiangcheng Biotechnology Institute, China).

### Real-time PCR

One piece of lung were harvested and stored at −80°C. Total RNA of these tissues was isolated with TRIZOL regent (Invitrogen, USA) and reverse transcribed into the first strand (cDNA) using DNase I, oligo (dT) 20 and Superscript II reverse transcriptase (Invitrogen, USA). The reverse transcription was conducted at 37°C for 15 min, 95°C 5 sec. Primers were designed with Primer 5.0 according to the gene sequence of mouse to produce an amplification product (Table 7). β-actin was chosen as the house-keeping gene to normalize target gene levels. The PCR cycling condition was 36 cycles at 94°C for 40 sec, 60°C for 30 sec and 72°C for 35 sec. The relative expression was expressed as a ratio of the target gene to the control gene using the formula 2^−(ΔΔCt)^, where ΔΔCt = (Ct_Target_-Ct_β-actin_)_treatment_-(Ct_Target_-Ct_β-actin_)_control_. Relative expression was normalized and expressed as a ratio to the expression in the control group.

**Table 7:** Primers used in this study

Genes	No.	Nucleotide sequence of primers (5′–3′)	bp
β-Actin	NM_007393.5	F: CCACCATGTACCCAGGCATT R: AGGGTGTAAAACGCAGCTCA	253
IL-1β	NM_008361.4	F: TGCCACCTTTTGACAGTGATG R: AAGGTCCACGGGAAAGACAC	220
IL-6	NM_031168.2	F: CCCCAATTTCCAATGCTCTCC R: CGCACTAGGTTTGCCGAGTA	141
IL-10	NM_010548.2	F: TAAGGCTGGCCACACTTGAG R: GTTTTCAGGGATGAAGCGGC	209
TNF-α	NM_013693.3	F: ATGGCCTCCCTCTCATCAGT R:TTTGCTACGACGTGGGCTAC	97
TLR4	NM_021297.3	F: CCATGCATTTGGCCTTAGCC R: AGAGCACTGAACCTCCTTGC	74
Myd88	NM_010851.2	F: GCTGGCAGGAGACTTAAGGG R: TCCGAGGGTTCAAGAACAGC	201
NF-κB	XM_006501106.2	F: GATCACACAGGCCGGACAAT R: CTCGGCTACACTCAGATCGC	156

F: forward; R: reverse.

### Statistical analysis

All data were analyzed by SPSS 17.0 software. Difference was tested by Ducan's multiple comparison test. Data are expressed as the mean ± SEN. Values in the same row with different superscripts are significant (*P* < 0.05).
